# Probing the complexity of wood with computer vision: from pixels to properties

**DOI:** 10.1098/rsif.2023.0492

**Published:** 2024-04-17

**Authors:** Mirko Lukovic, Laure Ciernik, Gauthier Müller, Dan Kluser, Tuan Pham, Ingo Burgert, Mark Schubert

**Affiliations:** ^1^ Laboratory for Cellulose & Wood Materials, WoodTec Group, Empa—Swiss Federal Laboratories for Materials Science and Technology, Überlandstrasse 129, 8600 Dübendorf, Switzerland; ^2^ Department of Computer Science, ETH Zürich—Swiss Federal Institute of Technology, 8092 Zurich, Switzerland; ^3^ Wood Materials Science, Institute for Building Materials, ETH Zürich, 8093 Zurich, Switzerland

**Keywords:** computer vision, machine learning, complex materials, pattern formation, wood properties

## Abstract

We use data produced by industrial wood grading machines to train a machine learning model for predicting strength-related properties of wood lamellae from colour images of their surfaces. The focus was on samples of Norway spruce (*Picea abies*) wood, which display visible fibre pattern formations on their surfaces. We used a pre-trained machine learning model based on the residual network ResNet50 that we trained with over 15 000 high-definition images labelled with the indicating properties measured by the grading machine. With the help of augmentation techniques, we were able to achieve a coefficient of determination (*R*^2^) value of just over 0.9. Considering the ever-increasing demand for construction-grade wood, we argue that computer vision should be considered a viable option for the automatic sorting and grading of wood lamellae in the future.

## 1. Introduction

Due to resource depletion and the challenges created by global warming and climate change, the demand for renewable resources such as wood has increased drastically [1,2]. Wood acts as a natural carbon sink, making it very appealing for the building industry to use wood from sustainable forestry as much as possible to reduce its carbon footprint. In 2019, for example, global building and construction works absorbed about 35% of the total energy generated and were responsible for about 38% of the total CO_2_ emissions, much more than any other industrial or transportation sector [3]. Therefore, the ability of trees to sequester CO_2_ from the atmosphere makes the long-term usage of wood and the materials derived from it, in construction for example, key for meeting sustainability targets in the transition towards a future circular eco-friendly bio-economy [4,5].

Although wood could potentially be used as a carbon sink in construction on a very large and global scale [2], we are far away from this scenario [1]. In Central Europe and other regions of the world, one of the main issues is that currently very few tree species are harvested for the construction industry. These are mainly conifers (softwoods) such as spruce (*Picea abies*) and pine trees. Besides being limited in terms of resources, this lack of variety favours the creation of monocultures, which are less resilient and at a higher risk of affliction compared to natural forests [6,7]. Moreover, spruce is particularly vulnerable to drier and warmer weather conditions provoked [7] by climate change [5]. Consequently, in Central Europe, we will have to start relying on a broader variety of tree species, especially hardwoods (deciduous trees), being more adapted to the expected climate conditions in the future [1,8]. In Switzerland, for example, it is projected that by 2080 low altitude spruce trees will almost disappear, while hardwood species like oak will become more prominent due to climate change [9]. Nevertheless, a shift towards hardwood tree species is complicated by the fact that the wood industry standards have been optimized for homogeneous softwood assortments. These standards [10,11] include wood grading and sorting, which means that new grading procedures will soon be required.

Solid wood is prepared for the wood and construction industries by sawing round wood (logs) into standardized sizes in the form of lamellae. These are then used as building blocks for manufacturing construction elements such as glued laminated timber (GLT/glulam) or cross-laminated timber. The lamellae must be graded and classified according to their mechanical properties into standardized strength classes. The grading process is usually automated by using machines that perform non-destructive tests to infer indirectly the tensile and bending strengths of each sample [12]. Various measurements are used to determine the indicating properties (IPs) related to the tensile and bending strengths of wood—properties such as wood density, knot density and modulus of elasticity. These IPs are then used in conjunction with a linear regression model to estimate the strengths of the lamellae [13]. Although the local density is important for inferring the strength, it is not enough. When dealing with large, industry-length, wood samples such as those in our case, there will likely be knots present. Anatomical characteristics, such as large single knots, knot clusters and fibre deviations, can cause a significant reduction in the strength of timber. The issue with the current grading methods in Europe is that they are standardized around a few wood species that are currently of relevance to the wood industry. Therefore, it is not straightforward to transfer them to other species.

**Figure 1.**
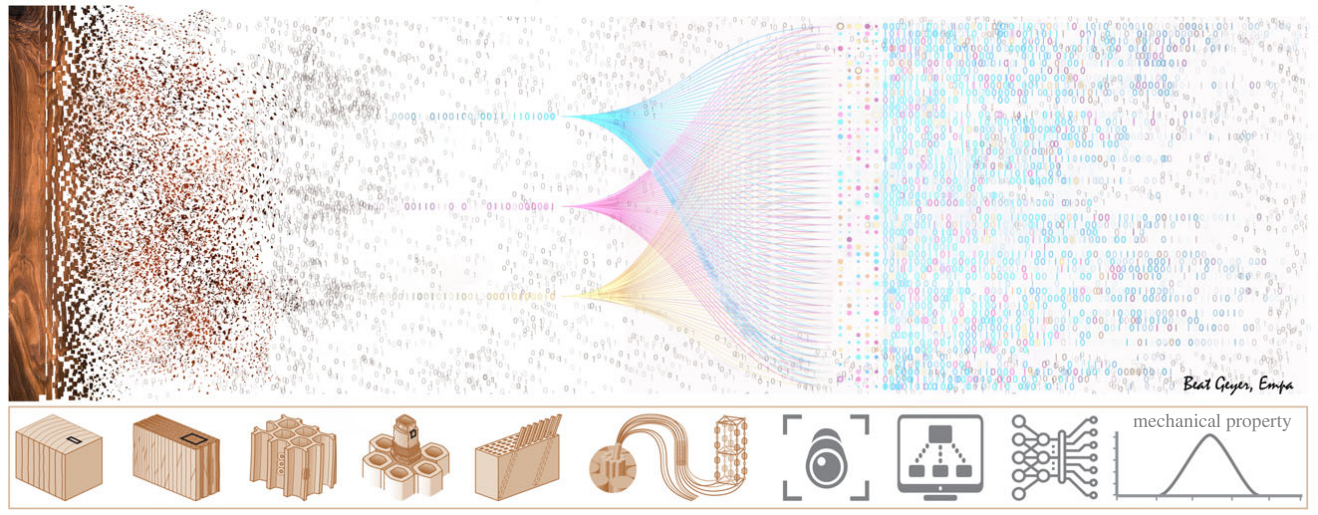
Hierarchical structure of wood. Thanks to emergent behaviour, the information regarding the mechanical properties of wood is ingrained in the complex fibre patterns observed on the surface. All elements of the figure are courtesy of the Empa graphics department. Some elements were also used by the authors of [14].

The main aim of our research is to develop a machine-learning model capable of accurately characterizing the mechanical properties of wood lamellae based on their characteristic surface patterns alone (figure 1). Such a model could potentially be implemented alongside current techniques used in the grading process of wood for the construction industry. In this paper, we present the results of the first step towards this goal, which is to predict the strength IPs of wood as measured by industrial grading machines, rather than the mechanical properties themselves. The reason is that training a model to predict the mechanical properties requires data produced on a large scale (of the order of thousands of samples) through the destructive testing of lamellae, which is very expensive and labour-intensive. The IPs, on the other hand, are readily available from automated non-destructive tests performed by dedicated machines in the wood industry.

We focus on wood lamellae from Norway spruce (*Picea abies*), which clearly manifest visible fibre patterns on their surfaces and can easily be captured by computer vision algorithms and analysed efficiently. The fibre patterns and orientation play a crucial role because they influence the strength, stiffness and overall performance of the material. Large single knots, knot clusters and fibre deviations significantly reduce the strength of timber. For lamellae with fewer and smaller knots, the influence and importance of other characteristics become more dominant, in particular, the local fibre direction (for details, see [15,16]). We discuss the role of complexity and the difficulties associated with it in light of existing tools available.

We use deep neural networks developed for computer vision and show that it is possible to determine the IPs of wood lamellae based solely on their images. Indeed, the reliance on IPs limits the accuracy of our model to that of the industrial machines in predicting the mechanical properties of wood. Nevertheless, we show that it is possible to take advantage of the complex fibre patterns, including knots, on the surfaces of sawn wood samples to train a neural network to predict properties that are known to be very indicative of the mechanical properties of wood. While the current industrial grading techniques use mechanical excitations and deformations of the samples, X-rays and knot density estimation as input to infer the various IPs, we focus only on colour images as input.

## 2. Fibres as building blocks

Wood is one of the most notable natural examples of a hierarchical complex structure [15]. It is made of cellulose, hemicellulose and lignin, forming a natural fibre composite at the cell wall level. Emergence in the context of wood refers to how the structural and mechanical properties of wood emerge from the arrangement and interactions of its building blocks [17]. Such a property makes wood a very robust and versatile biodegradable material and therefore attractive for the construction industry. However, the complexity and heterogeneity of wood make it extremely laborious to predict its properties, which in turn restricts its use in timber engineering and undermines its potential for enhanced future utilization. Mechanical properties of wood, such as strength, are tough to predict by simple mathematical laws as they can hardly be deduced from the collective properties of its individual components. Therefore, there is an urgent need to better characterize such systems, given its industrial application potential.

Common engineering materials such as metals, plastics or ceramics also have microstructure, but we do not consider them to be very complex. The reason is that their *representative volume element* (RVE) is very small compared to the linear dimensions of the structures or structural components they are used for. The RVE of a material is the smallest volume over which the average of mechanical or physical properties, such as Young's modulus, is representative of the whole. In a metal, the grain size may be of the order of 10 µm; hence, a volume of 0.1 mm^3^ will contain 10^5^ grains. In more heterogeneous materials such as glass fibre- or carbon fibre-reinforced composites, typical fibre diameters of 5–10 µm generally mean RVEs of the order of a few cubic millimetres. Therefore, the average value of a property over the RVE can be considered constant throughout the material [18].

Natural fibre-based materials such as wood offer an amazing array of hierarchies spanning from nanometres (cellulose microfibrils) to the centimetre level (wood tissue). The RVEs of the various substructures cover therefore a range from 10^3^ mm^3^ down to 10^−12^ mm^3^, i.e. fifteen orders of magnitude with several hierarchical levels: tissues, cells, cell walls, cell wall layers, cell wall biomacromolecules. Moreover, it is this overlap of substructures along different scales that is one of the main signatures of complexity and is common to most biological systems [18].

Emergent macroscopic structures of wood in general, such as fibre patterns, bigger rays, annual growth rings and knots, are the reflection of this complexity. Depending on various factors, including genetics, environmental conditions and tree developmental stages, the macroscopic structure of wood can vary greatly and produce specific macroscopic signatures [19]. We also know that these structures overwhelmingly account for the variability in mechanical strength of wood. Therefore, probing the complexity of wood is indispensable for understanding better the relationship between structure and properties. Indeed, a recent study showed that local fibre direction data, generated based on the analysis of the spindle pattern of bigger rays on tangential surfaces, could improve the prediction of the tensile strength parallel to the grain of European beech (*Fagus sylvatica*) lamellae [16]. In addition, Olsson *et al*. developed a novel strength grading method, approved for the European market, based on fibre orientation and dynamic excitation of Norway spruce lamellae [12,20]. Our research takes us further in this direction and presents a data-centric approach for deducing the mechanical properties, exemplarily shown for sawn spruce wood based solely on sample images.

## 3. From pixels to properties

Considering wood as an example of a hierarchical fibre-based complex system, we present here a data-centric approach based on machine learning for mitigating problems related to the high variation of mechanical properties. Indeed, even though the structure of wood and its physical properties have been studied for a very long time, there is an ongoing effort to develop a satisfactory multi-scale mathematical model for this task, which captures the entire complexity of the macroscopic structure. There exist numerical and statistical models, but they are usually computationally very expensive and are limited to a small range of scales [21]. The difficulty lies in the fact that the principles of structural organization at various length scales are still not understood well enough to formulate a model that would predict how the constituent elements interact and assemble into the emergent macroscopic structures observed [17,22]. Nevertheless, there do exist statistical models that are able to provide accurate predictions related to specific aspects of wood. Machine grading of wood, for example, is based on linear regression models. These models are derived from the tensile and bending strength test results obtained through the destructive testing of samples [13].

Thanks to the continuous increase in data availability, data-centric modelling has become a viable option and seems to be a promising approach in dealing with complex systems. An attempt in this direction is a method for generating finite-element models of wood lamellae from X-ray computed tomography scans [21,23]. The authors of this study used the tomography scans to determine the density distribution and reconstruct the geometry, pith, knots and local fibre orientations of board samples. They then created three- and one-dimensional finite-element models based on the reconstruction obtained to simulate the four-point bending behaviour. The results obtained using this technique are very encouraging, with *R*^2^ values of 0.83 for the bending strength and 0.93 for the global modulus of elasticity. Nevertheless, these results come at an extremely high computational cost and require the use of expensive computed tomography scanners.

In this article, we present a deep-learning approach that is *data-centric*, meaning that it gives more weight to data pre-processing and augmentation rather than the machine learning algorithm. The method presented in this paper is an alternative to common and effective methods. It is fast, reliable and comparatively inexpensive, and it works without relying on pre-existing mathematical models and ad hoc parameters. We made use of deep neural networks typically used in computer vision to construct a model that can link the emergent patterns and features visible on the surface of sawn wood with its macroscopic mechanical properties, including the modulus of elasticity and the bending strength. We used computer vision with transfer learning by adapting some of the more recent residual neural networks to process images of spruce wood lamellae obtained from the wood industry. Computer vision is an adaptable tool with the capacity to bring about significant improvements in many branches of the wood industry and industries related to it [24]. From a theoretical standpoint, the goal was to use spruce wood as an example of a complex system and to employ a purely data-centric approach to characterize its properties and establish a link between information and complex structure. We used Norway spruce because this was the only species of wood for which we could obtain enough data to train a deep neural network. From a practical point of view, we aim to progress further to a more versatile method that would allow the wood industry, especially small to mid-sized companies, to grade a more ample spectrum of wood species at a much lower cost and without the need for expensive scanners.

## 4. Methods

**Figure 2.**
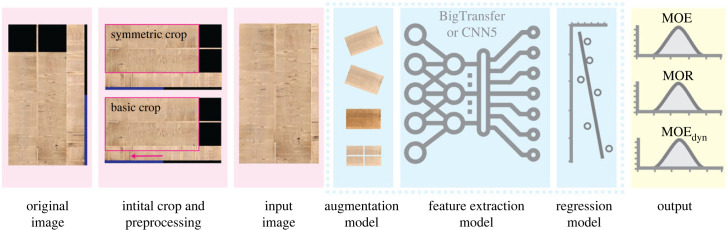
Schematic of our end-to-end pipeline. A raw image sample is used as input at the very left, which is passed through various stages of the pipeline until a vector of the 3 desired IP values is produced as output. The core of the pipeline is the pre-trained ResNet model represented by the middle box inside the dotted boundary.

The data available to us were in the form of standardized lamella images and structured data containing physical properties measured by an industrial wood scanner (Microtec's GoldenEye series scanner [25]). The spruce lamellae under study had a wood moisture content of 10 ± 2%, bulk density between 420 kg m^−3^ and 480 kg m^−3^ and dimensions of up to 25 cm in width and up to 5.5 m in length. More details are available in the last section of the electronic supplementary material. We could make use of a total of 15 104 images, all of which had the same layout, containing merged photographs of each face of a single lamella (figure 2; electronic supplementary material, figures S2 and S7). The images were of the same size with the same number of pixels, 1000 by 562. Since the average length of the lamellae is over an order of magnitude larger than the average width, the images of the faces had to be rescaled (compressed) to fit into a more readable aspect ratio. For this reason, the faces look distorted (compressed) in the figures. Consequently, the corresponding pixel sizes also changed.

The structured data were in the form of a table of various strength, stiffness and density IPs. We used only three of these IPs, namely the static modulus of elasticity (MOE), dynamic modulus of elasticity (MOE_dyn_) and bending strength, also known as the modulus of rupture (MOR). Each piece of timber is bent in the Microtec scanner (generally about its minor axis). The machine measures the stiffness of the piece and uses a correlation between stiffness and strength to assign a stress grade (see the standards DIN EN 408 : 2012-10 and DIN EN 338 : 2016 for details [10,11]). Since our goal was to predict the machine-extracted IPs by using images alone, we used the images as the input for our model and the three IPs as the labels for each lamella. The data were always divided into three sets used for training, validation and testing our models in the same ratio, 80 : 10 : 10, respectively.

The actual input images were cropped out from the original images in three different ways (see electronic supplementary material, figure S2). The two most successful types, which we call *basic* crop and *symmetric* crop, are shown in figure 2. The former produced input images with a resolution of 535 × 797 pixels and the latter a resolution of 410 × 797 pixels. All the original images in the collection contain the two black squares and many contain artefacts in the form of vertical saw cuts that are undesirable (indicated by the arrow in figure 2). Therefore, the cropping styles were chosen in a way that would avoid these cuts. In the case of the symmetric crop, the bottom segment was removed altogether. The basic crop maximizes the amount of information available by removing the minimum section of the bottom segment without changing the scale and without introducing any artefacts. Empirical evidence suggests that the artefacts noticeably distorted the input distribution and were therefore not helpful for the prediction task.

The setup that we used for training and testing our models is presented in the form of a pipeline in figure 2. Each model can be divided into three parts, each with a distinct task. The first part is responsible for the type of data augmentation. The second part is the backbone of the entire pipeline, and it consists of a modified ResNet model. It extracts meaningful representations of an image into a feature vector that is subsequently used for the prediction of the IPs of the lamellae. The final part is the regression model (regressor) for which we decided to use a multi-target approach. Given an input image, it requires a single model to predict multiple target variables simultaneously. Although this choice did not lead to clear performance benefits in our case, we decided to use it because it significantly reduced the required training resources and using multiple targets made the model training process more stable (electronic supplementary material, figure S1). The regression models (or regressors) can be a simple single layer with all-to-all connections or more sophisticated, such as a random forest algorithm.

At the core of our pipeline, we have a feature extractor, a modified version of the ResNet50 model, pretrained on the ImageNet-21k dataset, a bigger and more recent ImageNet dataset [26,27]. We will refer to this modified ResNet model as the *BigTransfer* model, in line with [26] (see electronic supplementary material for details). We chose ResNet50 for its performance success record and its accessibility in terms of ease of use and availability [27,28]. We employed transfer learning in our models with and without *fine-tuning* of the ResNet50 architecture. Fine-tuning implies that all the original weights of the pretrained model are unfrozen so that they can be modified during the training phase with the domain-specific data—wood images in our case. Initially, fine tuning of the *BigTransfer* network was always used during our search for the most accurate model. After that, we trained a few models without fine-tuning to determine the impact that it has on the accuracy of the models.

We tried to improve the accuracy of the predictions by using augmentation techniques in the form of random flipping, random rotation, random cropping, randomly adjusting the contrast, saturation and brightness, applying a Gaussian blurring filter, and adding Gaussian noise to the images. One of our biggest challenges was developing (approximately) label-preserving methods. The requirement of label preservation is essential because there was no simple procedure to modify our labels according to the changes made by the augmentation method. For example, the presence of knots drastically affects lamella mechanical strength. Therefore, applying a common augmentation method such as cropping could harm performance since we might remove knots without updating the labels accordingly. Consequently, the range of parameters selected for the augmentation methods had to be limited. We explored the trade-off between diversifying our dataset and minimizing the introduced mismatch between the augmented images and unmodified labels. Details regarding augmentation methods can be found in the electronic supplementary material.

Note that every unique combination of initial crop, augmentation methods, *BigTransfer* and regressors constitutes a different model, which must be trained and evaluated. We refer to the training and validation of each model as a unique experiment. In each experiment, the model is trained by starting with the same *BigTransfer* model (with weights corresponding to the pre-training phase only). Each of the models was trained using two further approaches. One was based on the BiT Hyper-Rule from [26], which is based on stochastic gradient descent and employs a learning rate scheduler. The other approach used the Adam optimizer [29], where we mainly tuned the learning rate. As a loss function for our regression task, we decided to use mean squared error due to its simplicity and the fact that not many outliers were present in our dataset. If this were to change, then it might be reasonable to explore loss functions that are less sensitive to outliers, such as the mean absolute error or the Huber loss function [30].

**Figure 3.**
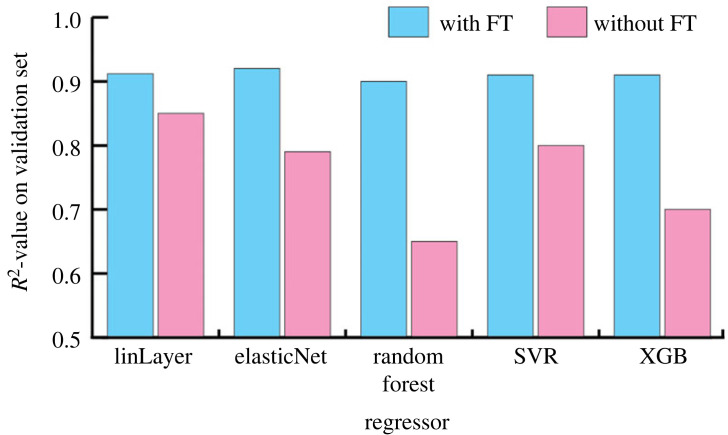
Model accuracy with different regressors on the validation set. We used the intermediate representation of our BigTransfer feature extractor as predictor for a single-target regression (MOE only). LinLayer at the leftmost represents the baseline. Each model was individually hypertuned using Optuna. Training on the fine-tuned representations consistently outperform their non-fine-tuned (without FT) counterparts. The standard deviation of each result was of the order of 0.01.

To improve further the accuracy of our models, we tried using different regression models in place of the last linear layer of the *BigTransfer* feature extractor (figure 3). When searching for the best regressor, we selected five of the more common regression models and used them to predict only one of the features, namely *MOE*, with and without fine-tuning. The regressors used were support vector regression (SVR) with a radial basis function kernel, *ElasticNet*, *RandomForest* and *XGBoost* regression. We hypertuned the most important parameters of each of the regressors to our multi-target task using Optuna [31] based on the validation *R*^2^. Once we were satisfied with a single regressor, we used it instead of the final layer of *BigTransfer* to predict our regression targets (MOE, MOR (*bending strength*), MOE_dyn_).

Finally, for the sake of comparison, we additionally used a simple neural network with five convolutional layers in place of *BigTransfer* as the feature extractor. We will refer to the former as the *CNN5* model. Each of its five layers consisted of a two-dimensional convolution sublayer, a two-dimensional max-pooling sublayer and a batch normalization sublayer. It served as our baseline for the performance validation of the *BigTransfer* feature extraction model. When comparing the two architectures, we always used the same pipeline with the same pre-processing methods, augmentations and regressors.

## 5. Results

In this section, we present the main results of the performed experiments. The intermediate results are shown in the electronic supplementary material. We start with the initial image cropping methods and input preparation. Three different models were trained using three different initial crops (see electronic supplementary material, figure S2) to determine which one produced the best results. We did not use any augmentations and the feature extractor was a ResNet50 model pretrained on the older and smaller ImageNet-1k dataset (we call this the *Transfer* model). The two best cropping methods, with similar performances, were the *Basic crop* and the *Symmetric crop* (electronic supplementary material, figure S3). Therefore, we discarded the *Double crop* in further experiments. Regarding the augmentation methods aimed at creating more data, we found that the best results were obtained when using a combination of flipping, rotations, colouring and cropping of the input images (details in electronic supplementary material, table Sii).

The most notable result of this study is that it is possible to predict the IPs related to wood strength using only on the surface images of lamellae as input. Since we were dealing with a regression problem, in all our experiments we used the coefficient of determination (*R*^2^) as the accuracy score of the model examined. Moreover, we repeated each experiment five times, using identical hyperparameters but with shuffling of the data. This gave us a more robust average score. In table 1, we present the results of the eight best-performing models. Each model was trained according to the pipeline in figure 2 with the hyperparameters indicated in the first column of the table and the *BigTransfer* as the feature extractor. The regressor in this case was a linear layer attached to the *BigTransfer*. As mentioned above, the *Double* *crop* was discarded and the augmentations, if used, were always the same, i.e. flipping with rotations, colouring and cropping. The most accurate model that we were able to train was based on input images with basic cropping, the Adam optimizer, a learning rate of 10*^−^*^5^ and the use of augmented data. It had a score of 〈 *R*^2^〉 = 0.903.

**Table 1.** Accuracy of different models (experiments) that also include the use of the two approaches based on the BiT Hyper-Rule and Adam optimizer. The *R*^2^ values are based on the output values obtained by using the validation dataset as input (see electronic supplementary material). The first column contains an identifier for each experiment. It consists of an abbreviation for the crop: BC for basic crop and SC for symmetric crop; followed by A if augmentations were used and NA if no augmentations were used. The last part of the identifier identifies the approach and the learning rate (lr) in the case where the Adam optimizer was used. We show the coefficient of determination, *R*^2^, averaged over five independent runs, for each of the three targets (IPs) individually [MOE, MOR, MOE_dyn_] (second column), which are then averaged to produce a single value in the third column.

model accuracy in terms of the coefficient of determination based on the validation dataset		
experiment	⟨*R*^2^⟩5 (Var[ *R*^2^])	⟨⟨*R*^2^⟩⟩IP (Var[⟨*R*^2^⟩])
BC, NA, BiT Hyper-Rule	[0.8481 (0.004), 0.8486 (0.003), 0.8359 (0.005)]	0.844 (0.004)
SC, NA, BiT Hyper-Rule	[0.8543 (0.001), 0.8521 (0.002), 0.8413 (0.002)]	0.849 (0.002)
BC, A, BiT Hyper-Rule	[0.8782 (0.001), 0.8729 (0.002), 0.8681 (0.002)]	0.873 (0.002)
SC, A, BiT Hyper-Rule	[0.8801 (0.001), 0.8738 (0.001), 0.8704 (0.002)]	0.875 (0.001)
SC, A, Adam lr = 0.0001	[0.8858 (0.006), 0.8793 (0.004), 0.8751 (0.005)]	0.880 (0.005)
BC, A, Adam lr = 0.0001	[0.8868 (0.002), 0.8801 (0.002), 0.8765 (0.001)]	0.881 (0.002)
SC, A, Adam lr = 0.00001	[0.897 (0.013), 0.8913 (0.012), 0.8881 (0.013)]	0.892 (0.013)
BC, A, Adam lr = 0.00001	[0.9074 (0.017), 0.9015 (0.015), 0.8989 (0.020)]	0.903 (0.017)

We tried to improve the score further by substituting the last linear layer of the *BigTransfer* with a more complex regressor. In the end, this did not bring about any improvement, so it was decided to keep the linear layer for simplicity. The results are shown in figure 3, which compares different regressors attached to *BigTransfer*. As mentioned in the Methods section, all the models were trained using fine-tuning of the *BigTransfer* neural network. However, we wanted to also determine the impact of fine-tuning on the accuracy, by applying a few models without using fine-tuning.

Even without fine-tuning, which implies training only the regression model, the representation provided by the pretrained *ResNet50* (*BigTransfer*) seems to be resourceful enough for the task at hand, as evident by the *R*^2^ test score achieved by *ElasticNet* and *SVR—RBF* without fine-tuning. The results in figure 3 show that for every estimator, the performance is significantly better on the representation extracted from the fine-tuned version of our model. This is to be expected since fine-tuning allows the weights of the neural network to be modified in response to our lamellae images. Interestingly, the model performs relatively well even without fine-tuning. This is because the model was pre-trained on single object-centred quadratic images that are part of the ImageNet project [32]. In addition, none of the regressors outperform the linear layer model with fine-tuning of *BigTransfer*, which uses an all-to-all layer as the regressor.

**Figure 4.**
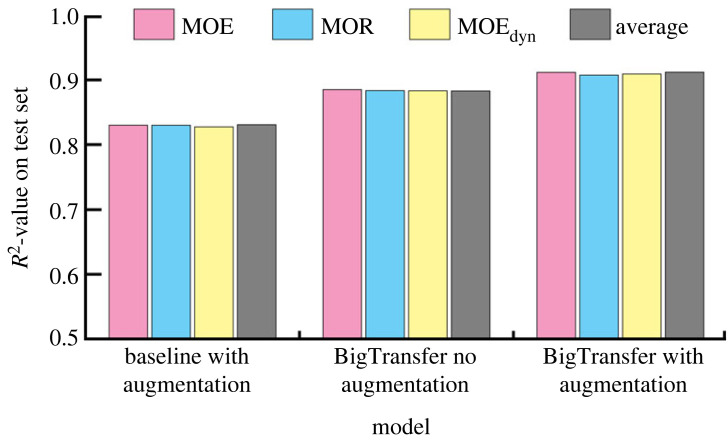
Overview of the performance of the BigTransfer model on test data compared to the baseline model. The coloured bars correspond to experiments where only one target was predicted. The value is an average over five different experiments. The grey bar corresponds to the average over the three targets and the values are: *R^2^* = 0.842 ± 0.005 for the baseline CNN5 model, *R*^2^ = 0.882 ± 0.002 for BigTransfer model without augmentation and *R*^2^ = 0.910 ± 0.003 for BigTransfer (for details, see electronic supplementary material, table Siii).

Finally, in figure 4, we show how our best model based on the *BigTransfer* feature extractor compares to a model that is identical except that it uses a CNN5 as the feature extractor and to another model that is identical apart from the fact that it does not use augmentation. We can conclude that models with the *BigTransfer* feature extractor perform much better than the baseline models and that augmentation slightly improves the accuracy.

Formally speaking, an *R*^2^ value of 0.912 implies that our best model can account for or *explain* 91.2% of the variability in the data. It is difficult to judge the quality of this result since we have no reference for direct comparison. In principle, we could use human level performance as a baseline. However, in this case, this is not a meaningful solution, because even for the most experienced sorters it is almost impossible to determine the mechanical strengths of wood lamellae solely via visual inspection. Nevertheless, the performance of our method is comparable to that achieved in [21], even though we used much less resources and a much simpler setup, i.e. only surface images.

## 6. Discussion and conclusion

Machine learning algorithms and neural networks can use pattern recognition to learn to make predictions from big data. In this study, we presented machine-learning models that could potentially be used together with the standard numerical models or even as an alternative for understanding better the complex relationship between structure and strength of spruce wood. By using computer vision algorithms to interpret the patterns formed on the surfaces of the wood lamellae, we were able to obtain good approximations of the strength-related IPs that we were testing for. The high accuracy of our models suggests that a recording and subsequent processing of data of surface patterns of the wood surface can sufficiently display the relationship between hierarchical wood structure and selective mechanical properties.

This is in line with the emergence property of fibre-based composites discussed by Jeronimidis & Vincent [18] and the results obtained by Ehrhart *et al.* in a similar study [16]. They used image analysis with predefined indicators of wood fibre directions to infer the strength of beech lamellae.

Our results show that it is possible to train a neural network to infer the IPs measured by industrial wood grading machines (Microtec's GoldenEye in our case) from simple colour images of the samples. The main contributing factor is the fibre direction and the patterns that emerge from it, which we believe can be exploited in other wood species for the same purpose. This was also the finding of [16], which studied beech wood. The authors characterized the local fibre orientations in lamellae using a single numerical grading parameter, which was then correlated to the tensile strength parallel to the lamellae using different machine-learning techniques. Therefore, it seems very likely that computer vision would work also for other tree species. However, it needs to be understood better to what extent the required information is present in the fibre patterns. This problem necessitates a systematic study on a wide variety of wood species, which is part of our ongoing research.

The lack of a perfect score of *R*^2^ = 1 (i.e. the variability unaccounted for) prevents us from concluding that all relevant information regarding the inferred values of the mechanical IPs of wood can be assessed via visible growth patterns. Nevertheless, the results are promising when it comes to the implementation of machine learning for the purpose of optimizing the use of wood starting from the initial production process. We assume that the accuracy of the results can increase even further, given more data. The results in figure 4 show that there is a slight increase in accuracy when data augmentation is used. This result becomes even more remarkable when we take into consideration that we are dealing with a regression problem and the challenge of maintaining the same label while modifying the image.

The method discussed in this article has the potential of simplifying the way sorting of wood lamellae is done in the future and may help establish a more data-oriented wood industry. To achieve this, a comprehensive destructive testing survey, which includes many samples of different tree species, must first be conducted. The results of the tests would provide precise information regarding the mechanical properties of wood, which could then be used as labels for training a computer vision algorithm that works with lamella images. A fast, simple, accurate, inexpensive and robust technique for predicting the strength of lamellae from images alone would result in a substantial increase in resource efficiency and would allow for *object-specific sorting*. The idea behind this proposed sorting technique is to be able to predict accurately the strength of a wood sample, independently of its shape, size and species, and identify instantly the type of application or object for which it is best suited. Since the machine-learning approach does not require pre-defined models or filters to be used, but rather constructs them based on the input data, it appears feasible to extend this method to other tree species with visible fibre patterns and enough data.

Before concluding, we present a few thoughts and ideas regarding the emergent properties of hierarchical complex systems, which are prevalent in nature and are becoming more and more prominent in the manufacturing industry in the form of fabricated materials [33–40]. These systems are characterized by many fundamental interacting components that evolve into emergent hierarchical structures and properties at larger special scales [41–43]. Due to these complex inherent interactions, it is often impossible to extrapolate to the properties of the entire structure by analysing only the elements at the smaller spatial scales. Nevertheless, there do exist methods that at least identify the presence of emergence and which try to quantify it. One such method, proposed in the publication by Hoel *et al*. [44], is based on a general measure of causation called *effective information* (*EI*), which depends on the size of the system's state space and reflects key properties of causation (selectivity, determinism and degeneracy).

The so-called *causal emergence* introduced by Hoel *et al.* implies that the macroscopic level (or spatial grain) with the highest *EI* is the one that is optimal to characterize, predict and retrodict the behaviour of the system. Furthermore, the search for the macroscopic level at which *EI* is maximal has a parallel in information theory: channel capacity is an intrinsic property defined as the maximal amount of information that can be transmitted along a channel at a certain rate [44,45].

**Figure 5.**
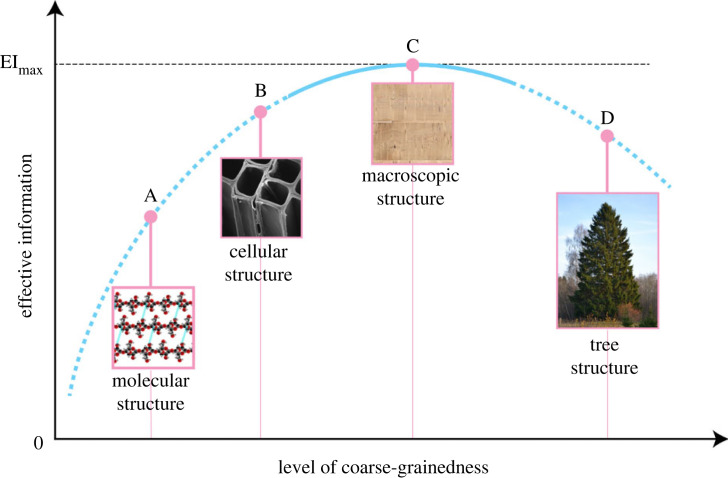
Characterization of self-organization and emergence relevant for the mechanical properties of wood lamellae. We conjecture that it is necessary to collect the structural information at the correct spatial scale to be able to determine the correlation between structure and the tensile or bending strength of wood accurately. In that case, it would be necessary to carefully analyse the fibre patterns observed on the surface of lamellae to determine their strength. This is all under the assumption that at this macroscopic scale, the information relevant for the strength prediction is maximized. Norway spruce tree insert by Ivar Leidus - Own work, CC BY-SA 3.0.

According to the prescription based on *EI*, we assume that the values of *R*^2^ can be mapped onto *EI* and that at the coarse grain level of our lamella images (point C in figure 5), we have $R^{2}=R_{\max}^{2}\approx 0.9$. If we use images with a lower resolution (i.e. higher grain size) to train the algorithm then the *R*^2^ values were always lower than $R_{\max}^{2}$. Increasing the grain level beyond that of C brings us to point D, which is the entire tree structure. At this point, in the absence of unique fibre patterns and fibre alignment, we believe that the accuracy of strength prediction decreases again.

Conversely, if we examine the sample at lower scales, such as the scale of the cellular structure of wood (point B) or even lower, at the molecular scale (point A), then we miss the emergent structures (e.g. fibres and their alignment, knots [46]) that are essential for inferring the mechanical properties. In principle, it would be possible to use X-ray computed tomography techniques with wood cell level resolution, such as the one used in [21], to reconstruct an entire 2 m wood lamella. However, the number of parameters, information and processing power involved make it very costly in terms of time and resources.

The sketch in figure 5 establishes a connection between our findings with those in [44]. It shows our estimation and interpretation of the dependence of *R*^2^ on the spatial scale adopted to study the complex system. Increasing and decreasing the resolution of the images used for training the machine-learning algorithm (uninterrupted part of the curve in figure 5) is equivalent to changing the grain size or macroscopic/structural level. When initially training our models with low-resolution data (for details see ‘Experimental setup’ in electronic supplementary material) to find good hyperparameters faster, we noticed that the *R*^2^ values always decreased when moving away from, but close to, point C. Therefore, we identify point C, not only as a local maximum on the uninterrupted curve but also as the global maximum of the entire curve. This leads us to formulate the hypothesis that the *R*^2^ used in this context is related to the *EI* as described in [44].

The example of Norway spruce wood lamellae indicates that machine learning could offer an alternative method for characterizing complex structures in wood. Transfer learning, particularly effective when transitioning between wood species models, holds the potential for enhancing existing sorting and grading methods and can become a valuable alternative that can refine current approaches. We also believe that it is timely to start discussions around interdisciplinary topics bridging natural structures, complex systems and artificial intelligence, to establish information theory, information transfer and data-science in the wood science community.

## Data Availability

The results that we present in our paper were obtained using images that were given to us from our industrial partner under the condition that we do not make them public. All the details regarding the model and the results are provided in the electronic supplementary material, everything except the images that we used to train and verify our machine learning model. A real sample of the images used is shown in figure 2. We can make the data available confidentially to a reviewer or a committee, but we cannot make it public. The data and code are available from the Zenodo repository at: https://doi.org/10.5281/zenodo.10782214 [47]. To access the data, please contact M.S. (mark.schubert@empa.ch). The data are provided in electronic supplementary material [48].
